# Natural products and drug discovery

**DOI:** 10.1093/nsr/nwac206

**Published:** 2022-09-29

**Authors:** David J Newman

**Affiliations:** NIH Special Volunteer, Wayne, PA 19087, USA

**Keywords:** artemisinin, glycopeptides, anthracyclines, dolastatin, diabetes 2, ACE inhibitors

## Abstract

This review covers the recent history of a series of very important natural products and their derivatives that are currently in use or under evaluation in the areas of anti-infectives, important cancer treatments that include antibody drug conjugates, followed by a discussion of type 2 diabetes (T2DM) drugs and angiotensin converting enzyme inhibitors. The current structures of the agents are shown, though in the case of some peptides used in T2DM drugs the standard single letter abbreviation for an amino acid is used.

## Teaser

So you thought that drugs came solely from the intellect of chemists and perhaps biologists. However as shown, Mother Nature is intimately involved in all these approved drugs.

## INTRODUCTION

This review demonstrates the utility of natural products in the development of drugs for human use, and explores how, over the years, natural-product-based drugs have led to very significant advances in the treatment of human diseases. The examples presented range from instances where the actual drug was a pure natural product, through to chemical modifications of natural products and products created in a research laboratory using details from the initial natural product. It should also be noted that fully synthetic drugs have been used in a number of disease areas where, to date, no natural product and/or derivative has led to a viable drug against those diseases. A listing of diseases for which, as of the end of 2019, there has not yet been an approved drug against that disease from a natural product source is given in the latest review by Newman and Cragg, published in 2020 [1], which is open access in the *Journal of Natural Products*. The areas that will be covered, in some cases in depth, in others not so deeply, include the following:

### Anti-infectives

This section covers the traditional Chinese medicine (TCM)-derived antimalarial artemisinin and its derivatives, which may have antitumor activity, with a description of the fermentation-derived precursor(s) and conversion to antimalarial agents. The antibiotic vancomycin (also known as a glycopeptide antibiotic) including both semisynthetic and totally synthetic variations that work against glycopeptide-resistant bacteria is also discussed in detail.

### Anticancer agents

This will include anthracycline-based molecules both naturally occurring and chemically modified, and will demonstrate their utility as agents against a number of cancers. Also, there will be a reasonably detailed discussion of the marine-derived dolastatins, now known to be from cyanobacteria, and how they have now become the ‘warhead of choice’ for a significant number of approved antibody drug conjugates (ADCs), including a recently approved Chinese ADC (Disitamb vedotin, or RC48). In addition, there will be an up-to-date discussion of the possibility that taxol may be a microbial product even though one current method of production is from plant-tissue culture. There will also be a discussion of other nominally plant-derived antitumor agents that may well have a ‘microbe in their background’.

### Metabolic diseases

Perhaps the most common disease that falls under this category is diabetes, both type 1 (T1DM) and type 2 (T2DM). The production of insulin by biotechnological means will be discussed and the history of this disease demonstrates the early knowledge from traditional medicine (both TCM and Ayurveda) over 2500 years ago that has led into agents mainly against T2DM that trace their ancestry to old herbal remedies. Included will be a discussion on the identification of peptidic agents from lizard venom that were modified and then led to a significant number of other peptidic agents (30–40 amino acids) that are now prime treatments against T2DM, and also other sets of synthetic compounds based on natural products that are now in general use against T2DM.

In addition, and also falling under the general term ‘metabolic syndrome’, will be a discussion of the drugs against high blood pressure based initially upon toxins from a Brazilian venomous snake. The first drug from that discovery, Captopril^®^, may well be the first rationally designed drug entity. But without the knowledge from the components of the snake venom, it would not have been designed and put into use in 1981.

## ANTI-INFECTIVES

### Artemisinin: initial discovery

The history of the discovery of a potential treatment for malaria, which came from ancient commentaries in TCM from details first written well over 2000 years ago, has been reported a fair number of times by many authors, but the definitive data are from You-You Tu's Nobel lecture in 2016 [2], and her more recent paper in 2019 [3]. The record of fevers that could be treated by TCM probably included descriptions of malaria, and dated back some thousands of years. Records from a similar period referred to the use of extracts from plants of the genus *Artemisia* (Chinese Qinghao) as medicinal agents.

Such extracts were initially mentioned as a specific remedy for what are now recognized to be descriptions of malarial symptoms in Ge Hong's *Zhouhou Beiji Fang* (or *Handbook of Prescriptions for Emergency*), which dates back to the Eastern Jin Dynasty (317–420 CE). Later works, such as the *Bencao Gangmu* (*Compendium of Materia Medica*) by Li Shizhen (Ming Dynasty, 1368–1644 CE), recommended application of ‘Qinghao and other techniques’ for relief of what were almost certainly malarial symptoms.

It was not until the discovery of the malarial parasites by Laveran in 1880, and mosquitoes as the vectors for avian malaria by Ross in 1897, and then for human malaria by Battista *et al*. by 1900 [4], that the true causes of malaria were identified.

What was a very significant comment in You-You Tu's Nobel lecture, was her realization that use of the conventional TCM methodology (hot to boiling water extractions) gave variable results. From searching more ancient literature covering use of TCM came her realization that room-temperature water extracts were the recommended method. Following that system finally yielded artemisinin (Fig. 1.1). Later work led her to use ether as the extractant, thus avoiding heat-induced loss. Using these techniques and knowing what was needed, she demonstrated that of the six *Artemisia* species known in China, only one, *Artemisia annua*, actually contained artemisinin.

**Figure 1.**
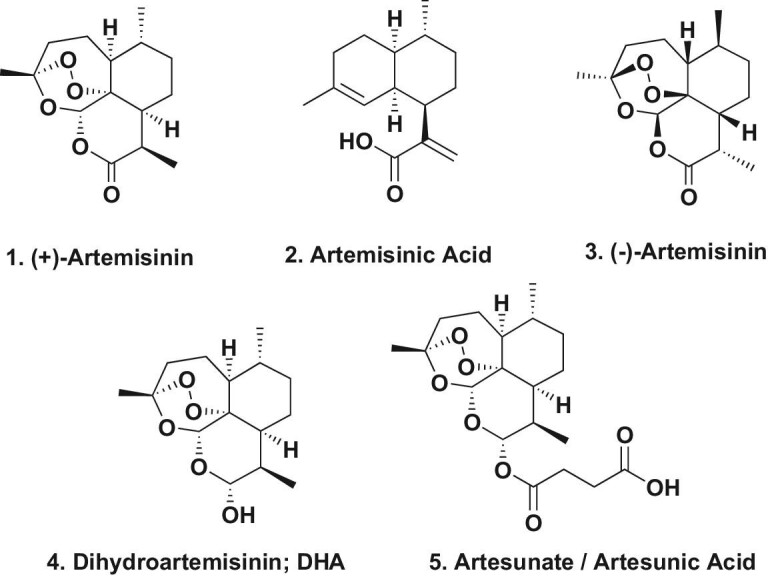
Artemisinin and initial derivatives; structures 1–5.

Inspection of the definitive structure of artemisinin showed why her group had very variable results in their earlier work, as the internal peroxide is a heat-labile group, and at above ∼45^o^C, the peroxo bridge would decompose. Her 2016 Nobel lecture article [2] contained only 25 references, however her 2019 paper was much more inclusive with 145 citations [3].

Further information as to her role and that of the others involved was published between these two papers in 2017 by Liu [5], and that report contained references to earlier work by Chinese investigators who had worked over the years with Professor Tu. However, a significant proportion of the references listed are not easily available to Western scientists as they are in Chinese journals and in Mandarin, however this paper is an excellent historical resource for Chinese investigators.

#### Artemisinin: production via biochemical engineering

Once this compound demonstrated its clinical superiority over the then conventional antimalarials, effectively all of which were loosely based upon various components of the chemical structure of quinine, the requirement for large amounts of the initial agent became a major problem that had to be overcome. Since the plant takes 90 to 120 days to grow from seed and was destroyed by the harvesting method of the time, either very large plantations, or methods to produce either the final compound or an intermediate, were necessary. The large plantation system is what was done in the USA under the auspices of the US Army in the 1980s, but it still required significant investment for low yields.

In 2004 the Gates Foundation funded initial work by the Keasling group at Berkeley, who were using modern biotechnological methods. This was followed up by its later commercial spin-off, Amyris, continuing the work. Amyris devised a process based on bioengineered S*accharomyces cerevisiae*, moving the necessary genes from *A. annua,* and then producing the essential intermediate artemisinic acid (Fig. 1.2) in large quantities. This was followed up by a semisynthetic chemical process that converted artemisinic acid into artemisinin [6,7]. The process was then licensed to Sanofi for large-scale production, with the ultimate aim of reducing the cost to $1 US per dose for use mainly by the World Health Organization. Just to demonstrate the number of entities that are interested in artemisinin and variations, the 2020 paper by Liu *et al*. [8] demonstrated the very substantial number of patents related to these compounds that have been awarded since 1968, thus demonstrating the value of Professor Tu's initial discovery.

#### Properties of chemically modified artemisinin derivatives

##### Stereochemistry of artemisinin.

The modification of artemisinin by groups in the West occurred almost from the beginning, as artemisinin, though active, had significant pharmacological flaws, particularly solubility. Similar work was certainly carried out in China in a similar time frame, but details were not available at the time in literature outside of China. Since the base molecule has stereochemistry, there was always the question as to whether or not there was a stereospecific aspect to artemisinin's antiparasitic activity.

The native molecule is the (+) antipode, so to determine if there was a stereospecificity in its antimalarial activity, and perhaps in other reported activities, the (−) antipode had to be synthesized *de novo* and then tested alongside its natural partner. Although there had been a number of synthetic routes to the (+) isomer, none had been reported on the (−) isomer (Fig. 1.3) before the 2018 paper by Krieger *et al*. [9]. In that report, it was shown that the antimalarial activity was independent of the stereochemistry. This was not expected, as most interactions with protein targets are stereospecific, and frequently the presence of the wrong antipode acts as an inhibitor of the desired interaction. The authors also emphasized that artemisinin is reported by different authors to bind to a significant number of different plasmodial proteins, thus casting doubt upon the hypothesis that artemisinin binds only to a specific plasmodial protein. In addition, both antipodes gave identical results within assay error against the *Plasmodium falciparium* parasite.

When tested for cytotoxic activity against CCRF-CEM leukemia cells and Adriamycin-resistant CEM cells, in both cases the (+) antipode was approximately twice as active as the unnatural (−) antipode, and since the standard error of the mean (SEM) values in the paired experiments did not overlap, these might be genuine but subtle differences, at least in those experiments. What is of significance is that the synthetic pathways that led to both antipodes can be easily modified to produce previously unreported compounds. Such compounds may well lead to molecules active against resistant parasites and perhaps certain tumor cells.

##### The activities of artemisinin and derivatives in multiple diseases

###### Antitumor activities of first-generation derivatives

Prior to 2004, there was evidence that the metabolite dihydroartemisinin (Fig. 1.4), which had been synthesized by Tu to confirm earlier findings on metabolism, might be a potential lead to a treatment for *Lupus-erythematosus*-related nephritis, since it inhibited the production of anti-double-stranded-DNA antibodies, with concomitant inhibition of TNF-α secretion and also the NF-κB pathway [10]. Though this paper was published in 2006, the work was certainly performed earlier, as mentioned by Efferth in a 2017 review [11]. In that review, Efferth provided an excellent series of tables that showed the published data (to the end of 2016) on the pharmacology of artemisinin (Fig. 1.1), dihydroartemisinin (Fig. 1.4) and artesunate/artesunic acid (Fig. 1.5). The latter compound was synthesized relatively early on in the investigations of artemisinin and was designed to overcome some of the metabolic problems found with the pure natural product.

As mentioned in the Efferth review [11], these compounds cover a wide range of potential interactions with mammalian cancer cells including, but not limited to, oxidative stress, induction of DNA lesions and arrest of the cell cycle, inducing various modes of programmed cell death, anti-angiogenesis and some interactions with essential signal-transduction processes.

As of the middle of July 2022, the NIH Clinical Trials database, having 146 studies listed using ‘artemisinins’ as the search parameter, only had three with any link to neoplasm treatment. Of these, two are in the USA, a Phase I (NCT03100045) and a Phase II (NCT04098744), with the third at the Phase I level in Germany (NCT00764036). It is possible that there are others in China that are not showing up in the NIH database, but in earlier years, Chinese trials were listed.

###### Activities of amino-artemisinin derivatives

Metabolism of the first generation artemisinins produced dihydroartemisinin (Fig. 1.4) as the initial metabolite upon treatment in humans. However, if a molecule did not have an oxygen group at position C^10^ then this metabolite would be avoided. In 2006, Haynes *et al*. [12] demonstrated that an artemisinin derivative with a cyclic nitrogen-containing group at C^10^ was active *in vivo* as an antimalarial and named the compound artemisone (Fig. 2.6). This compound was then modified by use of a piperazine substituent (Fig. 2.7). Subsequently this molecule was used as the base structure in order to produce the ferrocene-linked molecules (Fig. 2.8–10), which differed in the length and type of linkages to the ferrocene ring. An excellent review in 2020 by Xiao *et al*. [13] demonstrated not only the artemisinin-related compounds but other antimalarial agents that have been linked to this moiety.

**Figure 2.**
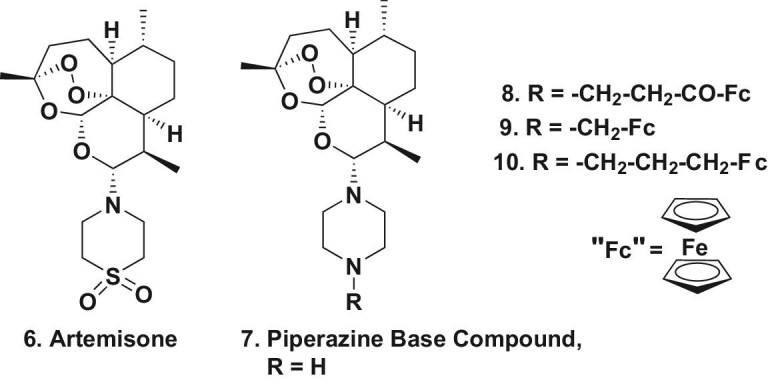
Amino-modifications and ferrocene derivatives; structures 6–10.

When tested against three *Pf* strains (two resistant and one sensitive) these molecules displayed excellent selectivity against the asexual blood stages of these strains. There was also some selectivity demonstrated on assaying them against human normal and tumor cells. These compounds still need further refinement but aptly demonstrate that such molecules have potential [14,15], with examples discussed in the next section.

###### Artemisinin-based compounds with antiviral and non-malarial antiparasitic activities

When chemically modified, the base molecule can exhibit a multiplicity of biological activities. These include activities against a variety of viruses and other parasitic diseases such as toxoplasmosis [16], with an extension to leukemias as a potential antitumor treatment. Many variations on the base molecule have been reported, however only a select few will be mentioned, but without listing their formal structures. Ho *et al*. from the National University of Singapore, in an excellent review in 2014 [17], included an excellent chart of reports from 1980 through early 2013. Another chart near the beginning of their paper demonstrated that ∼30%–40% of published reports dating from as early as 1982 extended the use of artemisinin(s) into pharmacological areas other than antimalarials.

From an antiviral perspective there is some reasonable evidence from *in vitro* studies for the activity of artemisinin and some of its derivatives against DNA viruses of the Herpesviridae and Herpesviridae, including human herpesvirus 6, herpes simplex viruses 1 and 2, Epstein-Barr virus and Hepatitis B virus. There were weaker activities against polyomaviruses and papilloma viruses, and little to no *in vitro* inhibitory activity reported for RNA viruses such as HIV 1 and 2, hepatitis C and influenza. Further information was given in a relatively recent review by Efferth demonstrating the antiviral effects of varied ‘first generation’ artemisinin derivatives, which is worth consulting in order to see the results from these agents [18].

###### Artemisinin dimers, trimers and dendrimers

Recent papers have demonstrated how relatively simple modifications (dimers and trimers of artemisinin) have antimalarial and other biological activities. A paper by Frolich *et al*. in 2018 [19] demonstrated the potential when such molecules were linked to rigid beads, and were then used to ‘catch’ the biological target(s), followed by identification of the ‘catch’ (captured protein(s) by mass spectroscopic techniques). That this was not a new technique was demonstrated by Laraia *et al*. in 2018 [20], but it identifies potential pharmacological targets of the chemical construct.

Artemisinin-based dimers and trimers with varying linkers between the molecules have been well described since the late 1990s and demonstrated significant activity against tumor cell lines, though the constructs frequently had significant toxicity against normal cells.

By using synthetic chemistry methods, the Frolich group [19] produced a number of molecules based on linkages to artesunic acid (Fig. 1.5). Of the 13 linked compounds described, the dimer (Fig. 3.11) and the trimer (Fig. 3.12) were investigated to determine if they bound to proteins in total lysates of human-HCMV-infected fibroblasts.

**Figure 3.**
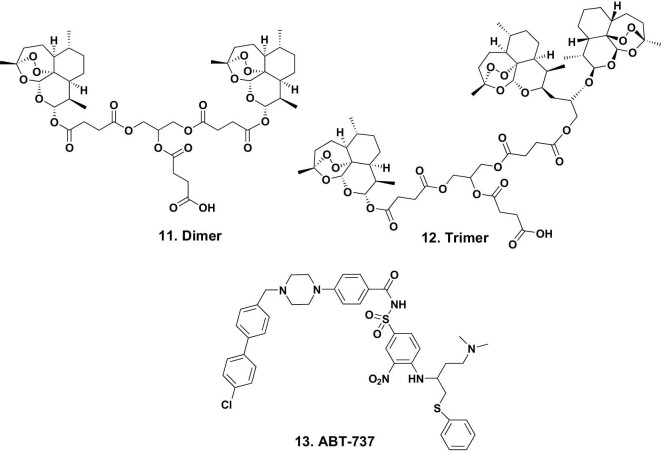
Artemisinin-based dimers/trimers and ABT-737; structures 11–13.

Table 3 in their paper demonstrated the numbers of both cellular proteins and HCMV-related proteins that bound. Whether or not these were all primary targets could not be determined from the initial experiments. However, the process can be repeated with timed infection experiments to further identify the pathway(s).

###### BAD, a human target of artesunate

In a paper in 2019, Gotsbacher *et al*. at Macquarie University in Australia, reported an unanticipated human target for artesunate (Fig. 1.5) as a result of the use of reverse chemical proteomics [21]. By using a phage expression system, displayed on a bacteriophage T7 vector that allowed unbiased interrogation of several human cDNA libraries, they reported that a probable human target of artesunate (Fig. 1.5) was the cell death promoter BAD. Under their experimental conditions, artesunate (Fig. 1.5) inhibited the phosphorylation of BAD, which then caused the formation of the proapoptotic BAD/Bcl-xL complex. This unexpected role of BAD might be utilized for clinical exploitation of artemisinin derivatives in the Bcl-xL life/death switch. The cytotoxicity of artesunate (Fig. 1.5) can be abrogated in HeLa cells if BAD is knocked down by siBAD. The 2012 paper by Watts and Corey [22] discusses ways to prove specific interactions by this technique. These data suggest that binding of artesunate is required for its apoptotic effect, and that its anticancer activity might well be independent of reactive oxygen species. Treatment with the Abbott drug candidate ABT-737 (Fig. 3.13), a known BH3 mimic that binds to various components of the Bcl-cascade, demonstrated synergistic activity in HeLa cells in the presence of artesunate. HeLa cells are constitutively resistant to ABT-737 due to high intrinsic levels of Mcl-1. This work opens up a whole new area for studying the interactions of artemisinin-derived compounds with human cell lines.

###### Synthetic peroxy compounds that reverse treatment expectations

After the initial discovery of the peroxy bridge structure of artemisinin, synthetic chemists, in addition to exploring the features of this natural product, began to synthesize potential compounds that had that particular motif or extended variations of it, as potential antimalarial agents. These led to a series of compounds, such as the trioxolane OZ277 (Fig. 4.14) based upon the 1,2,4-trioxane pharmacophore (Fig. 4.15) in artemisinin, plus others such as RKA182 (Fig. 4.16) based on a tetraoxane ring system.

**Figure 4.**
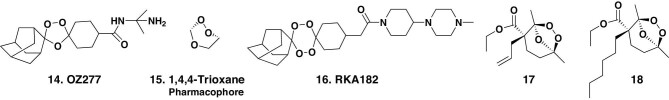
Synthetic peroxy compounds; structures 14–18.

In 2018, Coghi *et al*. [23] reported current work with such systems. They evaluated a set of peroxides that included bridged 1,2,4,5-tetraoxanes, bridged 1,2,4-trioxolanes and tricyclic monoperoxides for their *in vitro* antimalarial activity against *P. falciparum* 3D7 and antitumor activities in two human cell lines HepG2 and A549. In addition, the non-tumor cell lines Hepatic LO2 and bronchial BEAS-2B, plus the normal human fibroblast line CCD19Lu, were also used as toxicity controls.

The 26 compounds consisted of 11 tetraoxanes, 6 trioxolanes (3 sets of stereoisomers) and 9 monoperoxides, with artemisinin, artesunic acid, chloroquine (no structure shown) and taxol (no structure shown) as controls. In that study the synthetic ozonides exhibited high cytotoxicity *in vitro*, and selectivity. For example, ethyl (1R, 2R, 5S)-2-allyl-1,5-dimethyl-6,7,8-trioxabicyclo[3.2.1]octane-2 carboxylate (Fig. 4.17) demonstrated a selectivity index of ∼20, and ethyl (1R, 2S, 5S)-2-hexyl-1,5-dimethy-l6,7,8-trioxabicyclo[3.2.1]octane-2-carboxylate (Fig. 4.18) demonstrated a selectivity index of 28 against the HepG2 cancer cell line compared to the LO2 cell line. In contrast, artesunic acid (Fig. 1.5) had a selectivity index of 0.3 against the same cell lines. Finally, from an antimalarial perspective, all of the compounds tested were several orders of magnitude less active than the controls.

Interestingly, though designed around what were thought to be active antimalarial pharmacophores using data from a number of laboratories, these two compounds turned out to be potential anticancer leads with IC_50_ values between 360 and 590 nM against HepG2. To date, no further information has been published on these interesting results; they might lead to more novel antitumor agents in due course.

### Vancomycins

For a significant time period, vancomycin (Fig. 5.19) and then its close chemical relatives were frequently defined as the ‘antibiotic(s) of last resort’ due to their use when what could be considered simpler antibiotics, usually orally active, were ineffective against the Gram-positive infection. However, another significant problem was that vancomycin and later glycopeptides also exhibited significant nephrotoxicity, so their use had to be very carefully followed in patients. The pharmaceutical company Lilly originally introduced it into clinical medicine in the middle-to-late 1950s, though there is some debate in the literature as to the actual date. The actual structure was not fully defined until 1982 when the presence of asparagine within the peptide backbone was confirmed [24].

**Figure 5.**
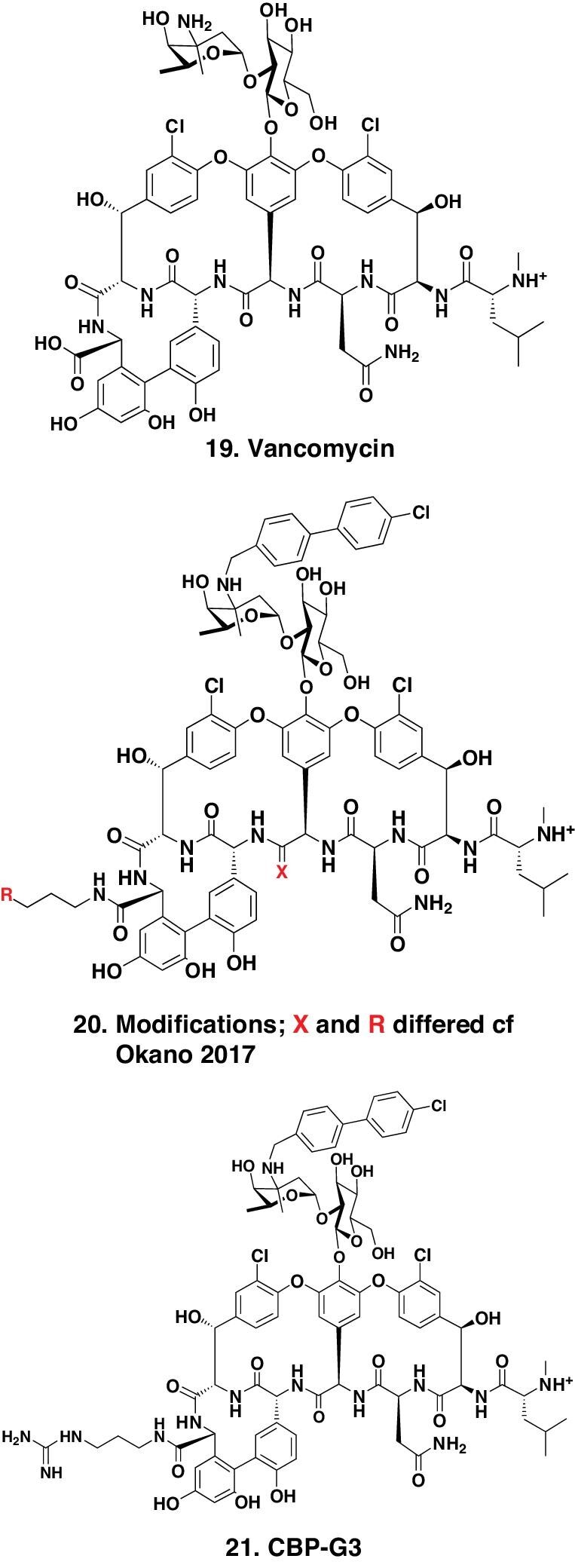
Vancomycin and synthetic derivatives; structures 19–21.

Resistance to vancomycin occurred relatively early on in its use, but the reason for the resistance was not known at the time as this occurred well before biochemical capabilities were available to pinpoint vancomycin binding sites, changes in bacterial cell walls etc. Once this capability became established in the 1970s, it became apparent that vancomycin (and its later naturally occurring or semisynthetic ‘chemical cousins’) inhibited the growth of bacteria by binding to the well-recognized ‘*L*-Lys-*D*-Ala-*D*-Ala-CO_2_H’ terminal sequences in the cross-links in the Gram-positive cell wall. Subsequent work demonstrated that the initial resistant phenotype *Van^R^* was due to the simple change of the terminal ‘*D*-Ala-CO_2_H to *D*-Lactate’ in the cases of the *vanA, vanB* and *vanD* clinical phenotypes. The terminal residue then changed to ‘*D*-Ser-CO_2_H’ for the other three clinical phenotypes *vanC, vanE* and *vanG*. Methicillin resistant (MRSA) Gram-positive microbes were also becoming resistant to vancomycin. The vancomycin resistance level due to the *D*-Lac modification was ∼1000-fold, and for the *D*-Ser modification, ∼140-fold compared to non-resistant microbial strains. Two terms that can often be seen in the vancomycin-related antibiotic clinical literature are VISA (vancomycin-intermediate *S*. *aureus)* and VRSA (vancomycin-resistant *S. aureus).* Depending upon the severity of the infection(s), other antibiotic classes were used to treat these infections, but not always successfully.

Interestingly, the widespread hypothesis that vancomycin resistance was due to the use of glycopeptide antibiotics in animal feeds was shown to be inaccurate by two reports: one in 2011 by D’Costa *et al*. [25] and the other from the same group a year later [26]. Both papers demonstrated that microbes isolated from deep core samples taken in Yukon ice fields, that from radiocarbon dating were over 10 000 years old, had similar resistance phenotypes to the current MRSA *S. aureus*.

In the years from 2009 to 2014, three semisynthetic glycopeptides entered clinical use in the USA and in some other countries, mainly in Europe. In 2009, telavancin, which was a close structural relative of vancomycin, was approved by the US FDA. This was followed in 2014 by their approval of two more ‘glycopeptidic antibiotics’: dalbavancin, which was derived from part of the known A40926 complex, and oritavancin, derived from chloroeremomycin.

Though not approved in the USA, teicoplanin, a mixture of closely related compounds, has been used in Europe for a number of years. Interestingly, some *VanR* phenotypes are not resistant to this mixture, though in general most strains are resistant to all.

#### Synthetic modifications of vancomycin

The laboratory that has been at the forefront of semisynthetic and synthetic work with vancomycin(s) is the Boger laboratory at the Scripps Research Institute in La Jolla, California. In the last 10-plus years, the Boger group has published synthetic chemistry papers showing how, by making ‘supposedly simple changes’ (nominally a simple change in one position within the peptide backbone) that though simple in concept required very clever and sophisticated synthetic chemical processes to achieve, a series of related vancomycin molecules that demonstrated very significant antibiotic activities against resistant MRSA and *E. faecalis* (both *VanA* and *VanB* phenotypes) could be synthesized. The Boger group then extended the synthetic chemistry process to other parts of the base molecule by adding relatively small ‘molecular parts’ from other glycopeptides in clinical use.

The compound structure (Fig. 5.20) was redrawn from the 2017 paper by Okano *et al*. showing the substitutions used [27]. The compilation of structural changes and their corresponding MIC tables in that paper demonstrated how these careful structural modifications ‘converted’ total resistance to *E. faecalis* and *E. faecium* (MICs of vancomycin ≥ 250 μg.mL^−1^) to molecules with MICs from 5 to 0.005 μg.mL^−1^ for these *VanA/E* resistant microbes.

Then in 2020, the same group published the results of utilizing different guanidino modifications on the C-terminus of vancomycin. These modifications improved the antimicrobial activity and exhibited a synergistic mechanism of action independent of *D*-Ala-*D*-Ala [28]. Using structure (Fig. 5.21) as the base, and then coupling a 4-chlorobiphenyl-methyl (CBP) modification, which they knew from earlier work, might give significant increases in activity. With X = O and R = a variety of guanidino substituents, they reported a series of relatively simple modified vancomycins that displayed sub-microgram activities (MIC levels) against significant vancomycin-resistant clinical specimens.

The following is a direct quote from that paper: ‘a prototypical member of the series, G3-CBP-vancomycin (*15)* exhibits no hemolytic activity, displays no mammalian cell growth inhibition, possesses improved and especially attractive *in vivo* pharmacokinetic (PK) properties, and displays excellent in vivo efficacy and potency against an especially challenging multidrug-resistant (MRSA) and VanA vancomycin-resistant (VRSA) *Staphylococcus aureus* bacterial strain.’

The structure (*15)* mentioned in the direct quote above from Wu *et al*. [28] is shown in Fig. 5, structure 21.

In addition to the papers referred to earlier, another recent paper from the Boger lab [29] gives an excellent precis of the modified vancomycin derivatives mentioned above, plus others from the Boger lab. They comment that these agents are now known in that laboratory as ‘maxamycins’. This paper is well worth reading to gain insight into the chemical modifications of a microbial product, which first saw ‘light of day’ in the middle-to-late 1950s, in order to overcome microbial resistance.

#### A comment on modern synthetic methods as route(s) to cGMP product(s)

**Figure 6.**
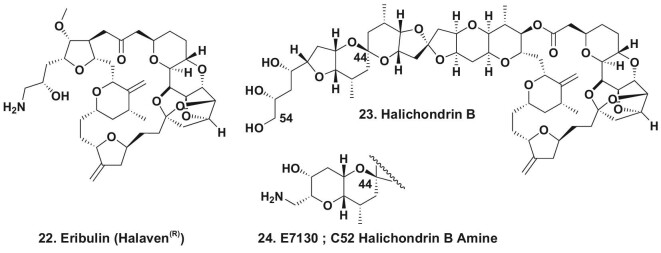
Synthetic halichondrin B derivatives; structures 22–24.

It should be emphasized that synthetic organic chemists have succeeded in the last few years in producing large quantities of cGMP-quality, partial or complete natural products that have become approved drugs, eribulin (Fig. 6.22) MW 730 being the prime example utilizing data from the Kishi synthesis of halichondrin B (Fig. 6.23) MW 1111. In addition, very complex potential leads to drugs can be synthesized from ‘scratch’ to give cGMP level compound in bulk, with the current best example being the total synthesis at the 10-gram level of a derivative of halichondrin B known as E7130 (Fig. 6.24) MW 1066. This was reported by the same Eisai group that synthesized eribulin under cGMP conditions [30]. This very complex modified natural product is currently in Phase I clinical trials in Japan, with the structures shown in Fig. 6.

## ANTITUMOR AGENTS

### Anthracyclines

These agents are aromatic type II polyketides that are assembled by sequential condensation of acyl-CoA units. They include, depending upon the carbon scaffold, molecules falling into structural classes covering anthracyclines, angucyclines, aureolic acids, tetracenomycins, tetracyclines and others. The first anthracyclines reported were the rhodomycins as antibiotics in 1950, but though subsequent molecules at times were reported to show antibiotic activities, their major ‘claim to fame’ was as antitumor agents.

The two best-known anthracyclines are the very close structural relatives, daunoubicin [31] (Fig. 7.25) and doxorubicin (Fig. 7.26). Random and mild mutagenesis was used as this was well before the identification of the producing gene clusters, so classical methods (UV irradiation, treatment with chemical mutagens etc.) were employed to obtain the more potent derivative doxorubicin (Fig. 7.26) [32]. Inspection of the structures of these two agents plus rhodomycin B (Fig. 7.27), nogalamycin (Fig. 7.28), aclacinomycin (Fig. 7.29) and steffimicin (Fig. 7.30), demonstrated that a common tetracyclic moiety was common to all, with the chemical diversity being due to tailoring enzymes working on carbohydrate moieties.

**Figure 7.**
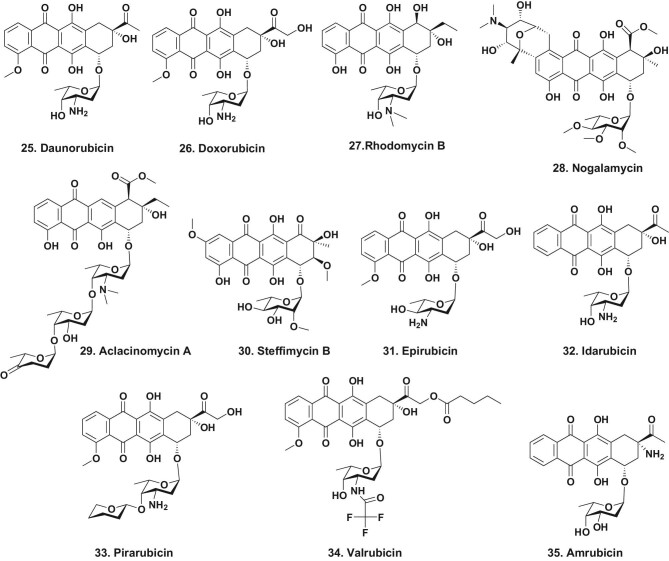
Anthracyclines; structures 25–35.

At the time of writing there are six different semisynthetic derivatives of daunorubicin (Fig. 7.25) in clinical use, doxorubicin (Fig. 7.26), epirubicin (Fig. 7.31), idarubicin (Fig. 7.32), pirarubicin (Fig. 7.33) and valrubicin (Fig. 7.34). In addition, the totally synthetic amrubicin (Fig. 7.35) is used in Japan. That entity contains a minimal version of the sugar daunosamine. All of these molecules have some cardiotoxicity and in general, treatment with these agents is limited to a number of treatment courses, often based upon the age of the patient [33].

Searching for novel anthracyclines is still ongoing. A recent review in *Natural Product Reports* by Hulst *et al*. [34] should be inspected by interested parties, but to date, none of the more recent compounds referenced in that review have become approved drugs, though work continues on genomic searching and modification by ‘mixing and matching’ gene clusters.

Anthracyclines were some of the initial molecules used as what can be considered ‘warheads’ linked to lipid carriers in attempts to overcome the cardiotoxicity referred to above, particularly in the treatment of breast cancer, with a pegylated-lipid version approved in various countries [35]. There is a non-pegylated version that is also approved for HER2-negative breast cancer. Looking at current modifications there is a very recent paper by Schettini *et al*. that demonstrates the potential of a non-pegylated liposomal doxorubicin to be a valid treatment for various scenarios in breast cancer treatment [36]. It should be pointed out, however, that no anthracycline linked to a monoclonal antibody (an antibody drug conjugate or ADC) has made it as a drug, though as will be seen in the next section, other natural products/derivatives have succeeded in becoming warheads on approved ADCs, initially against leukemias.

### Dolastatins

The story behind this class of compounds, which were originally isolated by the Pettit group at Arizona State University in the 1980s from the Indian Ocean sea hare *Dolabella auricularia,* is possibly the first example of how synthetic chemistry and early NMR techniques/HPLC were absolutely necessary for these compounds to be assigned their absolute structures. Once successful in both the structural assignments and in scale-up chemistry, though only at the sub-gram scale initially, they were then poised to become the progenitors of very potent agents that went into clinical trials (up to Phase II). Though they failed as single agents, they made their mark years later when slightly modified as warheads for ADCs, directed initially against leukemias.

The initial work of isolation and testing took many years and literally tons of the nominal producer. It demonstrated an ED_50_ of 46 picogram per milliliter levels against the murine leukemia P388 in *in vitro* assays, and a curative response against the same tumor in mice at ∼20 micrograms per kilo. Its flat structure was elucidated by NMR and MS studies in 1987 [37].

The base structure comprises an N,N-dimethylvaline (dolavaline; Dov) at P1, then a valine (P2), and three new amino acids, dolaleuine (Dil) at P3, dolaproine (Dap) at P4 and dolaphenine (Doe) at P5. However, the lack of any stereochemical information meant that the only valid method was total synthesis and to determine each center as the synthesis continued. The absolute configuration via total synthesis was published in 1989 [38], and a US patent was issued in 1990 [39]. Dolastatin 10 (Fig. 8.36) and its analogues bind to tubulin at the vinca alkaloid site, the same site as vinblastine, maytansine and phomopsin, and have a series of complex interactions with the machinery of the cell.

**Figure 8.**
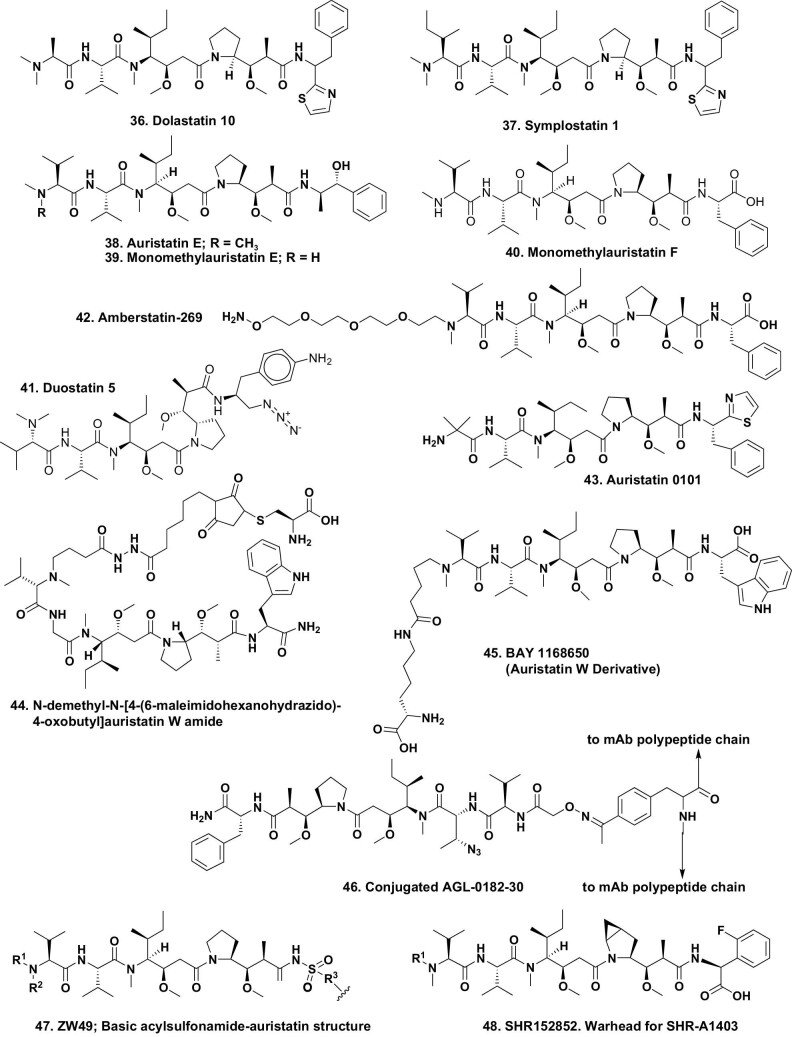
Dolastatin 10 and subsequent derivatives; structures 36–48.

As mentioned above, although a number of dolastatin analogues went into clinical trials as antitumor agents, none progressed beyond the Phase II clinical level. Where these compounds have excelled however, is as warheads for ADCs. Before these compounds became warheads, work was reported from the Moore laboratory at the University of Hawaii, who were working in conjunction with other marine natural product chemists, that demonstrated that dolastatins (and some very close relatives) were not produced by the nominal host animal but were in fact products of free-living cyanophytes, and were thus prokaryotic products. The first example was symplostatin 1 (Fig. 8.37), which was reported from *Symploca hynoides* in 1998 by Harrigan *et al*. [40]. This was then followed two years later by the isolation of dolastatin 10 from the cyanophyte *Symploca* sp., VP642 from Guam [41], where scientists had observed *Dolabella* species feeding on the cyanophyte. Later work from the Luesch laboratory and his collaborators found symplostatin 1 and dolastatin 10 from multiple *Symploca* sources [42]. In addition, the dolastatin 10 producing cyanophyte was taxonomically reclassified and ascribed to the new genus/species *Caldora penicillata* [43].

In 2002, a base patent was granted to Seattle Genetics scientists [44] covering a series of new pentapeptides that were based upon some of the earlier Pettit molecules. Specifically, these were two molecules based on the auristatin E (Fig. 8.38) nucleus, monomethylauristatin E (MMAE, Fig. 8.39), with the trade name of vedotin, and a close relative monomethylauristatin F (MMAF, Fig. 8.40), with the trade name mafodotin. Details were then published in 2003 on initial development of potent ADCs using these ‘warheads’ by Doronina *et al*. [45].

The first ADC based upon a dolastatin derivative, known generically as brentuximab vedotin, was approved by the FDA in 2011. This ADC had MMAE (Fig. 8.39) linked via a cleavable maleimide-based linker to the monoclonal antibody (brentuximab; cAC10), which was directed against the cell membrane epitope CD30 present in Hodgkin's lymphoma and anaplastic large cell leukemia. Since 2011 it has been approved for other lymphomas both in the USA and by the relevant agency in other countries.

A large number of pharma companies then entered into licensing agreements of one type or another with Seattle Genetics (known from the middle of 2020 as SeaGen, which may well be absorbed by a larger pharma company in the near future) in order to utilize MMAE/F (Fig. 8.39, 40) coupled to their own proprietary monoclonal antibodies, and utilizing cleavable or non-cleavable linkage methodologies.

Currently there are 14 ADCs that have received approval worldwide [46], with four utilizing MMAE (Adcetris^®^, Polivy^®^, Padcev^®^ and Aidix^®^) and one using MMAF (Blenrep^®^), thus readers can appreciate the very significant effect that these derivatives of the linear dolastatins have had on antitumor chemotherapy in the last 10–15 years. The impact of these natural products continues today with a significant number of ADCs at the early phases of clinical development.

Once MMAE and MMAF were shown to have significant antitumor activity, medicinal chemists in large and small pharmaceutical companies started to modify these base molecules, usually with the aim of discovering patentable molecules with similar activities. As of early 2021, the molecules shown below have been linked to proprietary monoclonal antibodies to treat different tumor types and are at various stages of preclinical and clinical trials in countries worldwide: Duostatin 5 (Fig. 8.41); amberstatin-269 (Fig. 8.42); auristatin 0101 (Fig. 8.43); N-demethyl-N-[4-(6-maleimidohexano-hydrazido)-4-oxobutyl]auristatin W amide (Fig. 8.44); BAY 1168650 (auristatin W derivative) (Fig. 8.45); AGL-0182-30 (Fig. 8.46), the basic structure for the mAb-warhead under the name ZW49 (Fig. 8.47); and SHR152852 (Fig. 8.48), the variant based on MMAE that is the actual warhead of SHR-A1403 [47,48].

Thus, from the initial findings by the Pettit laboratory in the 1970s, through the realization that dolastatins are bacterial in origin (from a free-living prokaryotic cyanophyte), coupled to the abilities of many medicinal/synthetic chemists spread world-wide, have come agents that when used as ‘warheads’ are revolutionizing the treatment of lymphomas and are now extending to other cancers. It should also be noted that though the dolastatins have been the centerpiece of this section, many other natural-product-derived antitumor agents are also being utilized as warheads. The number of approved agents shown in the 2022 paper by Fu *et al*. [46] demonstrates this point.

Older single agents derived from natural sources are now in use alongside the dolastatins, including maytansine, camptothecin precursors and pyrrolobenzodiazepines, all of which are well known from the 1980s or earlier; thus old agents have new leases of life.

### Taxanes

Taxol is well known for the treatment of breast cancer, and nowadays it is sourced either from needles from bulk growth of ornamental plants to yield the precursor 10-DAB III, followed by relatively simple chemical synthesis of the final product [49], or, particularly in Europe, via plant-tissue culture since the middle-to-late 1990s.

However, there have always been discussions since the early 1990s as to whether or not taxol production involved an endophytic microbe (probably a fungus). A relatively current paper that covers the possibilities is one by Kumar *et al*. in 2019 [50] demonstrating the differences between many fungal cultures and the levels of isolated taxol that have been reported.

A major problem with the levels reported over the last 30 years or so has been the lack of usage of old and well-known techniques of supplementation of microbial cultures, which were never formally published but were very well known in the 1950s to 1970s by scientists worldwide whose ‘job’ was to persuade microbes to produce secondary metabolites. Since there were relatively rapid movements between pharmaceutical companies by scientists involved in this area (the author being one of them) the methods were universally known and used, but as mentioned above, never formally published.

In a very recent paper by Daly and Cordell [51], the authors address the following points regarding fungal production of taxol. ‘Based on gene clustering in the producing fungi, it appears probable that the intact fungal pathway evolved initially in a particular way and was transmitted to other fungi and then to select plants in the genus *Taxus,* doing so on a geographically diverse basis. It will be interesting to observe the evolutionary relationships between the genomic data for the paclitaxel clusters from the different endophytes derived from diverse global locations.’

In addition, they list over 100 identified fungal endophytes that produce taxol at some level, that were isolated from 30 plant genera in 26 plant families, and they finish with the following comment. ‘Therefore, the extant view of plant origin first, fungal origin second, may well need to be reversed, at least in this instance.’

## METABOLIC DISEASES

### Angiotensin converting enzyme inhibitors

The renin-angiotensin system (RAS) can be traced back to a paper in 1884 reporting on the toxic properties of urine [52], then followed 14 years later in a paper discussing renin by Tigerstedt and Bergman [53]. Over the next 30-plus years, work by a number of investigators led to the identification of kallikrein in urine, leading to the unexpected discovery that neither kallikrein nor renin were vasoactive, but both released then-unidentified mediators from plasma. In the middle 1950s, Skeggs and colleagues reported (covered in a book in 1981) that renin liberated a decapeptide (angiotensin I), which is converted to the active peptide (angiotensin II) in the presence of chloride ions, by a factor found in horse plasma, which they named angiotensin converting enzyme (ACE) [54].

In the early 1960s, Ferreira, an associate of Rocha e Silva at the University of Sao Paulo, Brazil, joined the Vane laboratory in London (it should be noted that Vane had worked on the whole renin enzyme complex in earlier years). Ferreira and Rocha e Silva had shown in 1962 that the enzyme involved was a zinc metalloproteinase that could be inhibited by mercapto-derivatives [55].

In the Vane laboratory, Ferreira discovered that substances (now known to be small peptides) isolated from the venom of the Brazilian snake *Bothrops jararaca* not only potentiated the effect of bradykinin on smooth muscles, but also inhibited the inactivation of bradykinin. The first inhibitor identified was a pentapeptide (Fig. 9.49), which was a slow substrate of ACEs [56]. This report was rapidly followed by the isolation and then the total synthesis of the nonapeptide teprotide (Fig. 9.50) [57]. This molecule contained an ACE-resistant proline-proline C-terminus. To demonstrate how similar findings occur more frequently than is usually realized, in 1970, a Japanese group isolated a bradykinin-potentiating peptide from the Japanese snake *Agkistrodon halys blomhoffii* [58].

**Figure 9.**
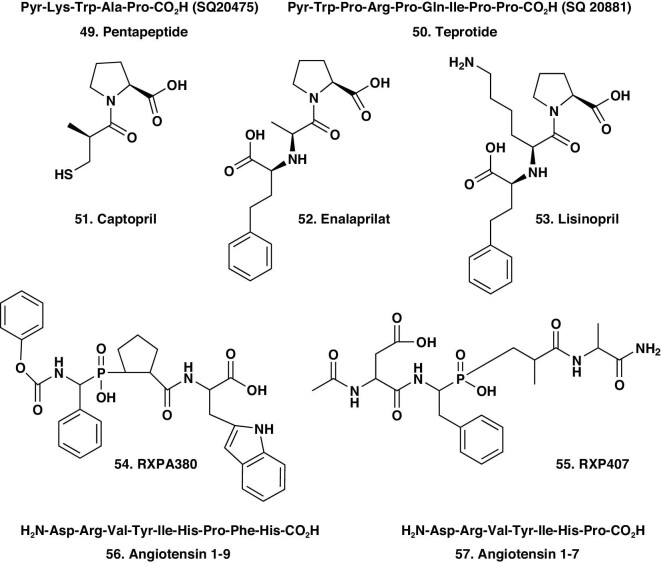
The derivation of ACE inhibitors; structures 49–57.

The data mentioned above from teprotide, coupled with data from other peptides from the snake venom studies in Vane's laboratory, became the scientific impetus for the synthesis of what can legitimately be considered the first rationally designed drug entity starting from a natural product. This drug from Squibb in the USA was approved under the name of Captopril^®^ (Fig. 9.51) for the treatment of hypertension in humans.

**Scheme 1.**
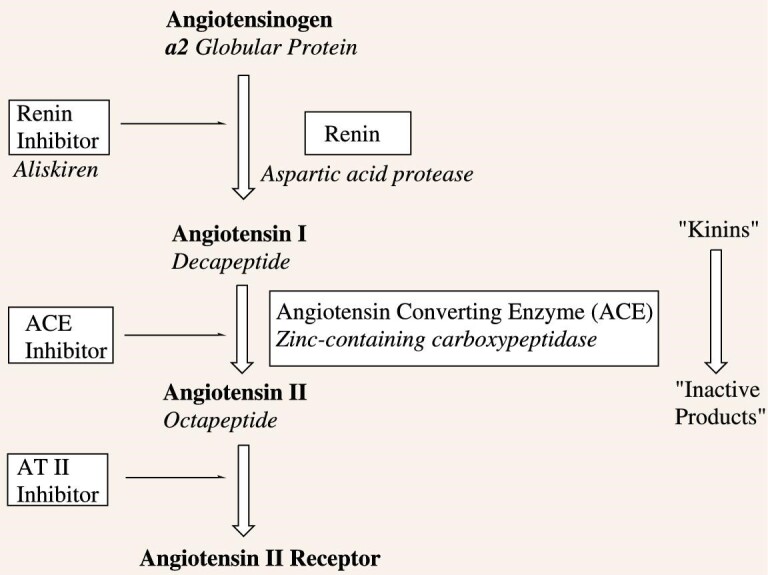
The angiotensin-renin cascade.

The group at Squibb initially chose to use carboxypeptidase A as their model for testing their synthetic molecules. This was a lucky choice, as later work on the crystal structure of the ACE ‘C-domain’ demonstrated that the three-dimensional structure is not related to carboxypeptidase A; rather it resembles the membrane metallo-endopeptidase (MME) known as neprilysin [59]. Since carboxy-peptidase A is actually a zinc-metallodipeptidase that functionally imitated ACE, all was well. Scheme 1 demonstrates the cascade that demonstrates the relationship amongst these enzymes/peptides in humans.

Following on from captopril, the work by Patchett *et al*. led to the development of enalaprilat (Fig. 9.52) and lisinopril (Fig. 9.53) [60]. Inspection of these structures definitively shows their ‘background in natural products’ since they resemble the tripeptides Phe-Ala-Pro and Phe-Lys-Pro respectively. Five years later, Patchett and Cordes published a significantly larger report of the design work and biology around the ACE-inhibitors, which is well worth consulting for information as to these discoveries [61].

In the same relative time frame, protein chemists/biochemists began to interrogate the structure of ACE and discovered that crystalline ACE had two internal areas of homology that covered ∼600 residues when their amino acid sequences were compared. These were named the ACE N-domain and ACE C-domain. They are ∼60% homologous when comparing DNA and amino acid sequences. If one compares simply their catalytic sites, then 89% homology is shown in these regions, but they exhibit different ‘affinities’ when measured by inhibition constants. Captopril (Fig. 9.51) was found to be modestly N-selective whereas the later series, enalaprilat (Fig. 9.52) and lisinopril (Fig. 9.53), were more C-selective. In contrast, the phosphinic tetrapeptide RXPA380 (Fig. 9.54) is 3000 times more selective for the C-domain versus its close phosphinic tetrapeptide relative RXP407 (Fig. 9.55), which is 1000-fold more N-domain selective [62]. Later work by Danilov *et al*. in 2011 demonstrated mutational relationships in mature human ACE [63].

However, in the last few years it has become obvious that there exists at least one other ACE, known as ACE2 [64,65]. This is an exopeptidase that catalyzes the conversion of angiotensin (Ang) 1 to the nonapeptide Ang 1–9 (Fig. 9.56) or the conversion of Ang II to Ang 1–7 (Fig. 9.57). ACE2 appears to be a chimeric protein formed by duplication of two genes and there are ancient orthologs found in the tunicate (sea squirt) *Ciona intestinalis* and in the primitive chordate amphioxus *Branchiostoma floridae*. Also of significant import from a disease perspective is that ACE2 was identified as the receptor protein for the SARS virus [66], and it is also the target of the SARS-CoV-2 virus from 2020 to date.

From the discovery, initially made by Brazilian pharmacologists and physicians, of the activity of small peptides in the bradykinin system, isolated from the venom of *B.jaracaca*, has come a series of extremely important antihypertensive drugs, but the discoveries have also led to a much more nuanced appreciation of what the original target protein was thought to do, and what it actually does in many biological systems. It should be noted that the protein is extremely ancient and has been found in one form or another in all taxonomic kingdoms.

### Diabetes

#### Type 1 diabetes

Childhood-onset type 1 diabetes (T1DM) is the disease that caused very significant numbers of deaths before the advent of, initially, porcine or bovine insulin, followed by the production of human insulin (Humulin^®^) by Lilly. The citation that shows the work in this effort is from a review published in 2021 by Riggs [67], and in that article there is the following significant quote: ‘For example by 2020 the genes for insulin can be made in a few hours by an automated instrument and then cloned and expressed by a single person in about a week.’

That is all that will be covered in this review on insulin as the modifications to the natural product are from the use of genomic techniques, not from the application of chemistry, even though in earlier days, modifications to bovine and porcine insulin did use classical peptide modification techniques. Potential treatments for what is known nowadays as ‘Metabolic Syndrome’ are also not included. This is a series of metabolic consequences that arise from mainly diet and lack of treatment (or deliberate non-treatment) of dietary decisions as to carbohydrate ingestion, non-treatment of high blood pressure, etc., which is linked to non-insulin dependent diabetes or T2DM.

### Non-insulin-linked treatments for diabetes, both T1DM and T2DM

Although insulin is the gold standard for T1DM and recent data have indicated that various insulin modifications may well be a newer potential treatment for T2DM, over the last 20 years or so, as some, though not all, of the ‘causes’ of carbohydrate metabolic changes that led to T2DM variations in patients have been and are being further identified, the pharmaceutical industries in a number of countries have begun to explore a variety of non-insulin agents as ameliorants of this multi-organ problem, with a significant number being based on natural products or modifications thereof.

#### Guanidines: agents against diabetes that began as a herbal remedy

There is one very well-known series of agents that were not covered in the first analyses made by the author and his colleagues, which covered the 1997-onwards reviews of drug sources as related to natural products but initially covering antitumor agents and anti-infectives.

The guanidines are directly descended from anecdotal usage of the perennial herb *Galega officinalis* Linn as a herbal remedy in the Middle Ages in Europe. Versions of the Culpeper herbal suggested that it had antidiabetic properties (effectively ‘sweet urine’). The complete Culpepper herbal as of 1850 is available as part of the ‘Project Gutenberg’ e-book series [68], but earlier versions are available in some national libraries as reference texts. In addition to this e-book, there are other much more recent articles that give information on the use of the natural product galegine (Fig. 10.58), which was used over centuries but not as the pure chemical, though there are reports in the French literature as late as the mid-1930s of usage of partially purified extracts as treatments [69].

**Figure 10.**
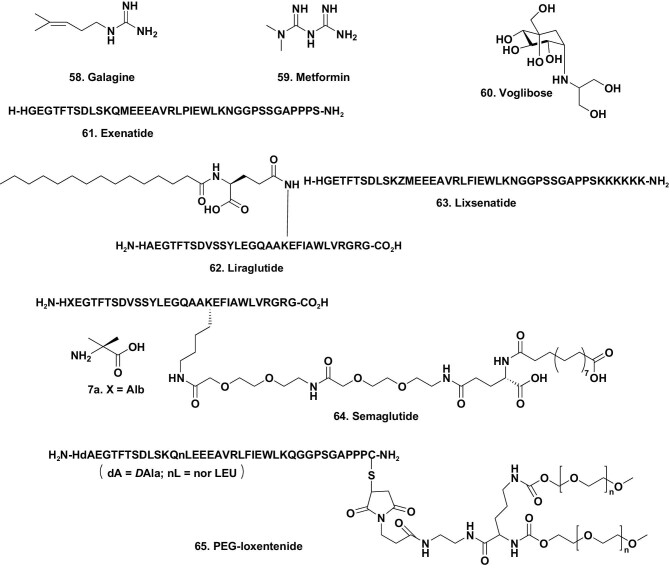
Galagine and GLP-1 agonists for T2DM treatment; structures 58–65.

The fundamental chemistry report on usage of guanidino compounds in what we now know as T2DM was a paper in the *Journal of Biological Chemistry* in 1918, in which Watanabe demonstrated that guanidino compounds, including simple derivatives related to galegine (Fig. 10.58), could reduce blood sugar levels [70]. The initial synthesis of metformin (Fig. 10.59) was published in 1922 by Werner and Bell [71]. However, though its glucose-lowering potential was published, it was not used as an antidiabetic agent at that time. In the early 1940s, metformin was ‘rediscovered’ as part of a search for antimalarial agents, and in 1957 the French physician Jean Steme first reported its ‘old’ potential for treatment of adult-onset diabetes (T2DM) [72]. Over the next few years, Sterne continued to publish on the utility and mechanism(s) of this agent, with the last paper published in 1964 before some later unreferenced book chapters [73].

From the reports of the potential of this class of compounds, in 1958, metformin was introduced in the UK and other European countries, but it was not for another 36 years, in 1994, that metformin was approved by the US FDA, where it was introduced in 1995; two reports at that time were key publications that confirmed the favorable risk/benefit ratio in the management of T2DM [74,75]. In 2017, Bailey published a review giving the history of metformin, and in that article, Table 1 is a timeline with excellent commentary demonstrating the path from 1772 to 2011, with relevant citations given at each major point in the story [76].

**Table 1.** Newman and Cragg codes.

Code	Brief definition/review year
B	biological macromolecule/1997
N	unaltered natural product/1997
NB	botanical drug (defined mixture)/2012
ND	natural product derivative/1997
S	synthetic drug/1997
S^*^	synthetic drug (NP pharmacophore)/1997
V	vaccine/2003
/NM	mimic of natural product/2003

From the perspective of using this compound as an antitumor agent, at the present time (August 2022) a search of the NIH clinical trials database (www.clinicaltrials.gov), with metformin as the drug search candidate, yields 72 Phase III studies using metformin as a potential antitumor drug. These are recruiting or are underway, with over 400 at the same trial level being completed.

#### Drugs other than guanides approved from 1997 to September 2019 against T2DM

In a series of reviews written from 1997 to 2020 by the author and colleagues [1,77–81], we analyzed the chemistry behind all drugs approved by the US FDA or comparable agencies in other areas of the world. We deliberately only listed/counted a compound once, irrespective of how many other countries or diseases it was subsequently approved in or for. The first review in 1997 [77] specialized in antitumor and anti-infective agents, but from the 2003 review we extended the reviews to include most diseases where drugs had been approved since 1981.

These reviews were designed to be cumulative with the last two in 2016 [81] and 2020 [1] being open-access articles in the *Journal of Natural Products*. In the 2003 review [78], we introduced definitions that subdivided the sources when chemical syntheses were used to produce an approved drug entity. These subsets were to better define molecules that required ‘bio/chemical forensics’ to decide if they were based on a natural product and/or were recognized by the biological system as a mimic. The basic terms are shown in Table 1.

From the 2020 review, which covered up to the end of September 2019, there were 39 non-insulin antidiabetic agents approved by at least one governmental agency worldwide. As of the end of September 2019, their breakdown was as shown in Table 2.

**Table 2.** Antidiabetic agent sources as of September 2019.

N	ND	S	S/NM	S^*^	S^*^/NM
1	8	4	16	1	9

The single natural product in the above table is voglibose (Fig. 10.60), an α-glucosidase inhibitor first isolated in Japan in 1981 from *Streptomyces hygroscopicus* var *limons* and approved in 1994 in Japan for treatment for T2DM. Two other natural product derivatives (structures not shown), acarbose and miglitol (under ‘ND’ codes), were also approved in 1990 and 1998 respectively, targeting the same enzyme system.

##### Modified peptides that are incretin mimics (GLP-1 agonists).

In 2005, the peptide extenatide, or Byetta^®^, a 39-residue peptide (Fig. 10.61), was approved for T2DM treatment. This agent is classified as a glucagon-like peptide 1 (GLP-1) agonist, and it was based on one of the peptides in the saliva of the Western USA lizard known colloquially as ‘Gila monster’ (*Heloderma suspectum*). It is also known as an incretin mimic since GLP-1 is the naturally occurring incretin hormone.

From 2005 a number of other GLP-1 mimics, all based upon modifications of the extenatide base skeleton, have been approved, including liraglutide (Fig. 10.62) in 2009, lixisenatide (Fig. 10.63) in 2013 and semaglutide (Fig. 10.64) in 2017. A non-natural amino acid known as Alb (Fig. 10.7a, **X = Alb**) was inserted to avoid degradation. A pegylated version, PEG-loxenatide (Fig. 10.65), was approved in 2019 with two unusual amino acids inserted, the ‘*D*’ isomer of alanine and *nor*leucine. All of these are used for treatment of T2DM, working as GLP-1 agonists, and fall under the ND category.

##### Agents against dipeptidyl peptidase IV.

**Figure 11.**
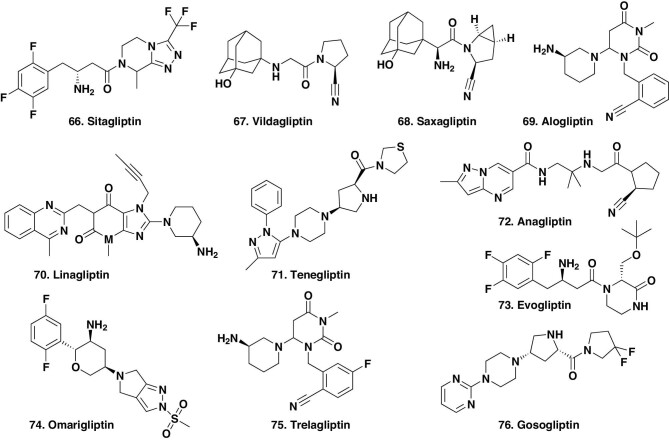
DPP-IV inhibitors for T2DM treatment; structures 66–76.

In T2DM treatment, another current target is DPP-IV (dipeptidyl peptidase IV), and the agents that target this enzyme all fall under the S/NM category. Eleven compounds directed against this target were approved between 2006 and 2016, with their structures shown in Fig. 11 starting with sitagliptin (Fig. 11.66) in 2006. After this initial agent, over the next 10 years agents were approved as follows: vildagliptin (Fig. 11.67) in 2007, saxagliptin (Fig. 11.68) in 2009, alogliptin (Fig. 11.69) in 2010, linagliptin (Fig. 11.70) in 2011, and teneligliptin (Fig. 11.71) and analgliptin (Fig. 11.72) in 2012. Then, in 2015, three more ‘gliptins’ were launched, evogliptin (Fig. 11.73), omarigliptin (Fig. 11.74) and trelagliptin (Fig. 11.75). In 2016, the latest to date was gosogliptin (Fig. 11.76) following approval in Russia.

##### Agents against the sodium-dependent glucose transporter.

Currently there are nine sodium-dependent glucose transporter (SGLT-1/2) inhibitors with structures based upon the non-selective natural product phlorizin (Fig. 12.77). This led to dapagliflozin (Fig. 12.78) in 2012 and canaglifloxin (Fig. 12.79) in 2013. Then, in 2014, four compounds using this base structure were approved: empagliflozin (Fig. 12.80), ipragliflozin proline (Fig. 12.81) and tofogliflozin (Fig. 12.82), with the last one in 2014 being luseogliflozin (Fig. 12.83), which contained an unusual thio-sugar. A gap occurred until 2017 when ertugliflozin (Fig. 12.84) was approved. It should be noted that this compound structure is close to that of the 2012 dapagliflozin (Fig. 12.78).

**Figure 12.**
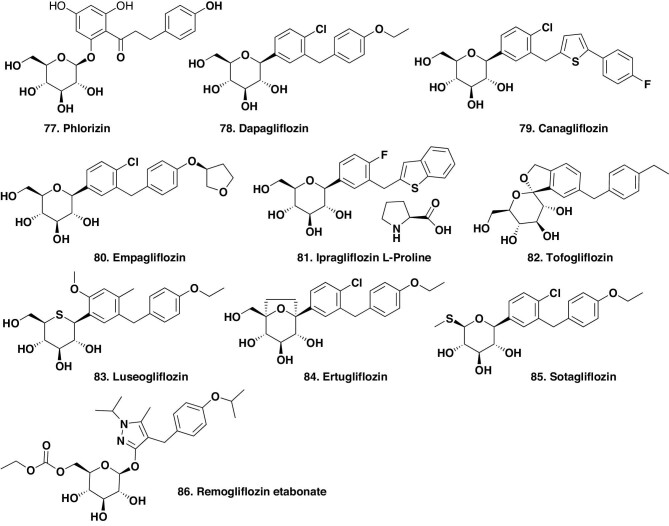
SGLT-1/2 inhibitors for T2DM treatment; structures 77–86.

In the first nine months of 2019 (the end date of our last published review) two more of this class of compounds were approved: sotagliflozin (Fig. 12.85), which also closely resembles dapagliflozin (Fig. 12.78) but with a methyl sulfur substitute in the sugar moiety in place of the normal hydroxyl group, and remogliflozin etabonate (Fig. 12.86).

The ‘gliflozins’ that are targeted against this protein complex fall under the S^*^/NM code. In addition, a recent paper by Shaffner *et al*. [82] covers the pharmacology of these inhibitors in detail and should be consulted in addition to the translational medicine aspect of these agents, which was covered by Beitelshees *et al*. [83].

##### Post-September 2019 drug approvals.

Since the publication of the 2020 review [1], as of the end of July 2022, the following three agents have been approved/launched for the first time (Table 3). Please note that their actual dates of approval might differ from their launch date in a specific country and that only the first approval and/or launch is noted.

**Table 3.** Agents approved 2021–2022.

Name/year	Mechanism	N & C codes	Structure #
Imeglimin/2021	GSIS enhancement	S^*^/NM	Fig. 13.87
Zegalogue/2021	Glucagon receptor agonist	ND	Fig. 13.88
Tirzepatide/2022	GIP/GLP-1 agonist	ND	Fig. 13.89

One of the three drugs is a small molecule exhibiting a novel triazine structure, Imeglimin (Fig. 13.87). It has activities similar to metformin and can best be considered as a cyclic metformin derivative [84]. From the natural product aspect, the other two are peptidic in nature and are direct agonists of specific receptors, with zegalogue (Fig. 13.88) being approved in 2021, and the other, tirzepatide (Fig. 13.89), in 2022. The details of producing tirzepatide (Fig. 13.89) on a kilogram scale were published by the Lilly scientists involved in 2021, and demonstrate the methodologies necessary to proceed from a lab scale of less than a gram to kilogram quantities under cGMP conditions [85].

**Figure 13.**
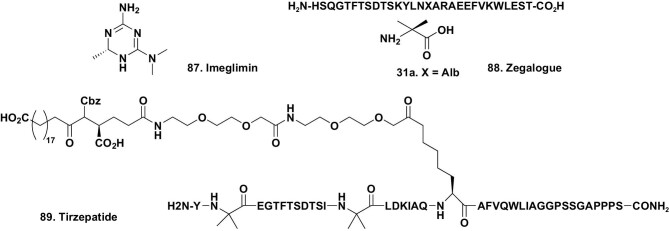
2021–2022 agents for T2DM treatment; structures 87–89.

##### Methods of T2DM peptidic drug design.

In 2022, Wang published a paper on the design parameters that were used to develop tirzepatide (Fig. 13.89). This discussion is an excellent primer on how to design complex peptidic drugs [86]. In the article, Wang identifies a website (www.comparediabetesdrugs.com) that compares drugs used in the treatment of both T1DM and T2DM. It is an excellent resource for scientists interested in this class of drugs.

## CONCLUSIONS

What is extremely interesting from a natural-product-chemistry perspective, is the progression from medieval herbal remedies (Goat's rue aka *Galega officinalis* Linn), via chemistry, to metformin (Fig. 10.59) and other derivatives that were developed from another early natural product, phlorizin (Fig. 12.77), which have been, and in most cases still are, in use as oral treatments for T2DM. Due to major recent advances in peptide synthesis, the two agents (zealogue and tirzepatide) referred to above in Table 3, which were approved for use in 2021 and 2022 under the ‘ND’ code, may well become prototypes for molecules in the future, directed against specific organelles.

## A NOTE ON STRUCTURES

All structures were drawn using the program ChemDraw (v.19.1) and then inserted into the text. Due to their size, in certain cases, polypeptides had significant numbers of amino acids (20 plus) together with non-peptidic sidechains such as fatty acids and/or polyethylene glycol (PEG) substituents. The structures used the single letter codes corresponding to the international usage for *L*-amino acids within ChemDraw. Details of this nomenclature are shown at the following URL: https://www.fao.org/3/Y2775E/y2775e0e.htm. If there were modifications of amino acids used, they are shown under the relevant base figure.
